# Supplementary material for: The interactome of intact mitochondria by cross-linking mass spectrometry provides evidence for coexisting respiratory supercomplexes
Source: Mol Cell Proteomics. 2017 Dec 8;17(2):216–32. doi: 10.1074/mcp.RA117.000470 (PMC5795388; doi:10.1074/mcp.RA117.000470)
Supplement: Supplemental Data [file supp_RA117.000470_133922_0_supp_23978_fzffwf.zip › spectra_annotation/mito_spectra_annotation/36-2-6-1-4-1.pdf]

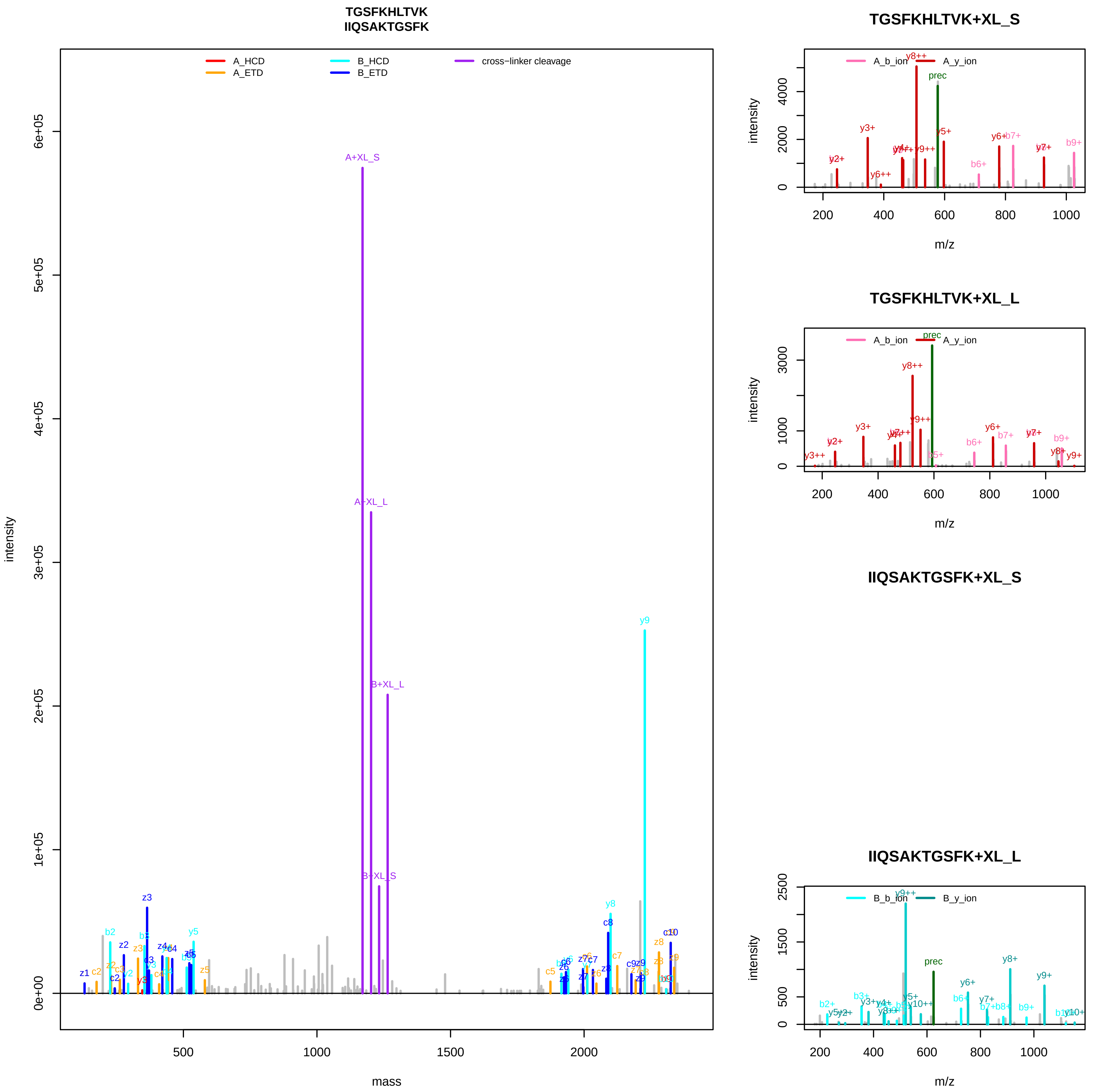

TGSFKHLTVK
IIQSAKTGSFK
A_HCD
A_ETD
B_HCD
B_ETD
cross−linker cleavage
A+XL_S
A+XL_L
B+XL_L
B+XL_S
y9
y8
c8
z3
b2
y5
c10
intensity
mass
0e+00
1e+05
2e+05
3e+05
4e+05
5e+05
6e+05
500
1000
1500
2000
TGSFKHLTVK+XL_S
A_b_ion
A_y_ion
prec
y8++
y3+
y5+
y6+
b7+
b9+
b6+
y2+
y6++
y7+
intensity
m/z
0
2000
4000
200
400
600
800
1000
TGSFKHLTVK+XL_L
A_b_ion
A_y_ion
prec
y8++
y9++
y3+
y6+
b7+
y7+
b9+
b6+
y2+
y3++
y8+
y9+
intensity
m/z
0
1000
2000
3000
200
400
600
800
1000
IIQSAKTGSFK+XL_S
IIQSAKTGSFK+XL_L
B_b_ion
B_y_ion
y9++
prec
y8+
y9+
y6+
y7+
b6+
b2+
b3+
y5+
b9+
intensity
m/z
0
500
1500
2500
200
400
600
800
1000
